# CD58; leucocyte function adhesion-3 (LFA-3) could be used as a differentiating marker between immune and non-immune thyroid disorders

**DOI:** 10.1007/s00580-018-2657-x

**Published:** 2018-03-03

**Authors:** Nadia El Menshawy, Mohammed Eissa, Hanaa M. Abdeen, Enas M. Elkhamisy, Nabil Joseph

**Affiliations:** 1 Clinical Pathology Department, Hematology Unit, Mansoura Medical School, Mansoura University, Mansoura, Egypt; 2 Clinical Pathology Department, Faculty of Medicine, Zagazig University and King Khalid University, Zagazig, Egypt; 3 Biochemistry Department, Mansoura Medical School, Mansoura University, Mansoura, Egypt; 4 Internal Medicine Department, Specialized Medicine Hospital, Mansoura Medical School, Mansoura University, Mansoura, Egypt; 5 Community Medicine Department, Mansoura Medical School, Mansoura University, Mansoura, Egypt

**Keywords:** Autoimmune thyroid disorders, Regulatory T cell, CD58, CD4/CD8, N/L ratio

## Abstract

The link between Graves’ disease (GD) and Hashimoto^’^s thyroiditis (HT) has been debated for decades due to the shared pathological and immunological components. Immune intolerance and inappropriate immune reaction against self-thyroid cells are distinctive features of both diseases, but definitive data for the clinical presentation of autoimmune thyroid disease remains unclear. To analyse the expression of T-regulatory cells, CD58, the CD4/CD8 ratio and the neutrophil/lymphocyte ratio and to determine if these parameters could be used as differentiating markers between auto- and non-immune thyroid diseases, 75 patients were enrolled in this study—40 with autoimmune thyroid disease (HT and GD ), 15 with non-immune thyroid disease, and 20 healthy controls. Multicolour flow cytometry was used to analyse CD58, T-regulatory cells (Treg) expressing CD4, CD25, HLA-DR and CD8 using different stained fluorescent labelled monoclonal antibodies. The neutrophils and lymphocyte ratio was also measured. Lower expression of Treg with higher expression of CD58 (LFA-3) was found in the autoimmune diseases when compared with the non-immune and control groups. ROC analysis showed that CD58 with sensitivity 88% and specificity 100% with cut-off value more than or equal to 29.9 indicates Hashimoto’s disease, while lower value indicates colloid goitre, and higher or equal to 29.84 indicates Graves’ disease and lower indicates colloid goitre with 100% sensitivity and specificity. CD58 could be used as differentiating marker between immune and non-immune thyroid disorders.

## Introduction

Autoimmune thyroid disease (AITD) is an immunological, clinically heterogeneous, specific organ disease typified by immune intolerance and inappropriate immune reactions against the thyroid gland (Weetman 2003). It can be presented as hypothyroidism, e.g. Hashimoto’s thyroiditis (HT), or as hyperthyroidism, e.g. Graves’ disease (GD).

GD is the predominant AITD and is caused by autoantibodies to thyroid-stimulating hormone receptor (TSHR) augmenting its action and inducing thyrotoxicosis; however, two other autoantibodies, directed to thyroid peroxidase (TPO) and thyroglobulin (Tg), may also occur and share classical marker of HT (Fountoulakis and Tsatsoulis 2004). Histologically, GD may exhibit mild lymphocytic thyroiditis and progress to hypothyroidism with clinical manifestation similar to HT (Ehlers et al. 2012). Clinical presentation of both diseases may overlap with non-immune colloid goitres and therefore present problems with regard to diagnosis, even requiring invasive techniques such as fine needle aspiration or even biopsy to reach the final diagnosis.

The association between GD and HT has been debated for decades as they represent opposite sides of the same coin. However, the pathophysiological relationship between TSHR, TPO and Tg autoantibodies remains uncertain (McLachlan et al. 2007).

Histologically, both GD and HT show thyroid lymphocytic infiltration which suggests the possibility of similar underlying pathogenesis. The complex interaction between genetic susceptibility and environmental factors initiating this process, and loss of immunological tolerance at multiple levels, explains how this interaction operates (Dantus 2008; Tomer 2010).

T-regulatory cells represent one of the most important immune regulatory cells in humans; they have a suppressive function on self-tolerance and therefore play a central role in the pathogenesis of autoimmune disease. There are different types of these cells with different expression markers and there are many differing theories regarding the role of these cells in different autoimmune and malignant diseases. Several studies on these cells focused on the expression of CD4, CD25 and HLA-DR (Nada and Hammouda 2014; Fountoulakis et al. 2008).

Development of tolerance is a complex process that includes central and peripheral mechanisms acting in concert to eliminate self-reactive lymphocytes (Armengol et al. 2008); however, T cell deletion by central tolerance may not eliminate all self-reactive cells. Another powerful mechanism involves regulatory T cells (Treg), such as naturally occurring CD4/CD25 (IL-2 receptor-chain) T cells, that control autoreactive effector T cells in the periphery which escape from thymus negative selection (Miyara and Sakaguchi 2007). The suppressive function of CD4/CD25 T cells is mediated by cell-cell contact (Sakaguchi 2005), while CD8/CD122 T cells (another type of naturally occurring Treg cells) operate by secreting a regulatory cytokine IL-10, not by cell-cell contact (Endharti et al. 2005).

Treg cells represent a specific lineage of immune regulatory cells in both humans and animals and, through their suppressive functions, have central role in the maintenance of immunological self-tolerance. Different varieties of Treg have been identified, some with CD4, CD25 and HLA-DR, other express CD4, CD25 and FOX3 (Curotto de Lafaille and Lafaille 2009). Other populations express transcription factors called Helios (Thornton et al. 2010).

The cell adhesion molecule, lymphocyte function-associated antigen-3 (LFA-3), also known as CD58, plays a central role for naïve and memory T helper cells during the early phase of an immune response. The LFA-3/CD2 pathway initiates strong antigen-independent cell adhesion, expansion of naïve T helper cells, and induction of large amounts of IFN-gamma in memory cells (Wingren et al. 1995). The release of IFN-gamma may upregulate expression of ICAM-1 and B7 on antigen presenting cells (APC) allowing multiple adhesion pathways to magnify the immune response; this permits transition from an autocrine to paracrine immune response. Co-expression of B7/LFA-3 provides an optimal APC function and enables a potent T cell response to minute amounts of antigens (Wang et al. 1999).

Several researchers focused on the value of neutrophil/lymphocyte ratio (NLR) in different cancers; however, little has been published about this predictor as simple, applicable, routine investigation to be used as a diagnostic tool in immunological thyroid disorders (Baykan et al. 2015; Ozyalvcli et al. 2014).

It is clear that the immune system plays a vital role in the control and progression of many diseases including immune disorders and cancers.

## Aim of study

This study was carried out to assess the expression of adhesion molecule LFA-3 (CD58), T-regulatory cells harbouring CD4, CD25 and HLA-DR along with CD4/CD8 ratio and N/L ratio in different types of thyroid disorders and to determine the applicability of these parameters as differentiating markers between autoimmune and non-immune thyroid diseases.

## Patients and methods

**Fig. 1:**
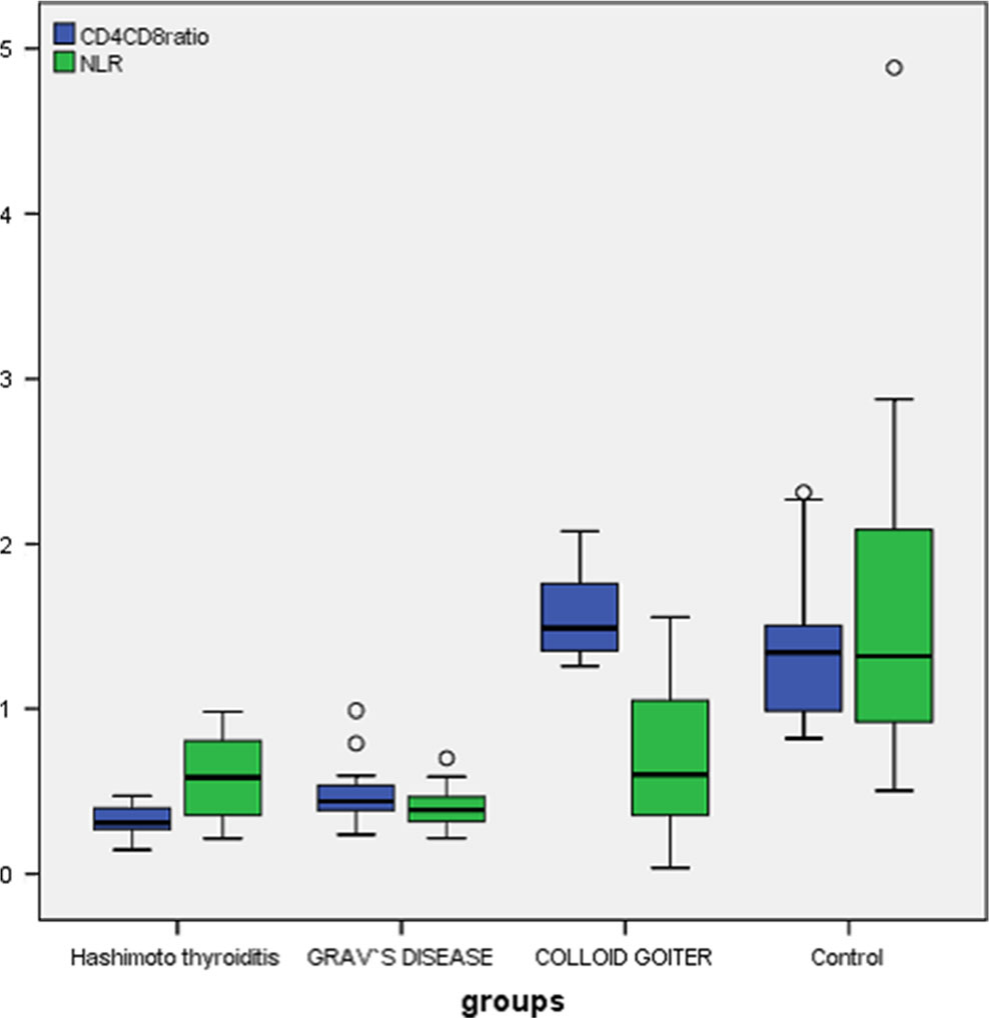
CD4/CD8 ratio (blue box) with highly significant difference among studied group. Hashimoto’s (0.326 ± 0.92), Graves’ (0.49 ± 0.19), and Colloid (1.56 ± 0.26) while normal healthy control (1.37 ± 0.45). Furthermore, *N*/*L* ratio (green box) was lower in Graves’ disease than Hashimoto’s, while higher in Colloid and normal control

This case control study was carried out from September 2013 to December 2014 at Mansoura Specialised Medicine Hospital, Egypt. There were 75 patients enrolled onto this study, classified into four groups: group I, (*n* = 25) Hashimoto’s thyroiditis (3 males and 22 females); group II, (*n* = 15) Graves’ disease (seven males and eight females); group III, (*n* = 15) colloid goitre (CG) (2 males and 13 females) as a non-immune control disease; and group IV, (*n* = 20) healthy, age matched (9 males and 11 females) as control reference (Fig. 1).

All patients in groups I, II and III were newly diagnosed and untreated at the time of blood collection. Patients were allocated into their group based on their clinical expression of AITD and the stage of thyroid dysfunction supported by commonly accepted clinical, laboratory criteria and radiological assessment, including neck ultrasound, Doppler flow in the inferior thyroid artery as well as Tcm99 thyroid uptake. Occasionally, fine needle aspiration and cytology of thyroid cells were also used to diagnose thyroid diseases. Patients assigned to group IV did not have a goitre and had no history of any abnormal thyroid dysfunction, surgical operation or any medical illness; all investigations for thyroid function were normal with no evidence of thyroid antibodies at time of study.

Venous blood was obtained from all patients and controls in the morning. Full blood count was performed on automated blood cell counter analyser Cell Dyne (Abbot), and any patients with evidence of inflammation or on immune modulatory drugs, or drugs known to intervene with thyroid function, were excluded from this study. Informed consent was obtained from every patient according to local ethical committees MFM-IRB/16.06.44.

## Methods

Measurement of thyroid hormones and thyroid autoantibodies by commercially available kits (DPC, Los Angeles, CA, USA) was used for estimation of serum-free triiodothyronine (FT3), free tetraiodothyronine (FT4), thyroid stimulating hormone (TSH), anti-thyroglobulin (anti-TG) and anti-thyroid peroxidase (anti-TPO) antibodies with the automated immunoassay analyser Immunolite 2000 (DPC Ltd., Gwynedd, UK). Measurement was based on solid-phase chemiluminescent assay.

Multicolour flow cytometry analysis (*BD FACSCAN II*) was performed using different fluorescent monoclonal antibodies labelled with fluorescein isothiocyanate (FITC), phycoerythrin (PE) and peridinin chlorophyll protein (PerCP)-conjugated anti-human monoclonal antibodies to measure control Ig1/g2a (anti-isotype control negative), CD4-FITC, CD4-PerCP, CD8-PE and CD8-FITC. The flow cytometer was calibrated before assay to check every laser beam and the accuracy of apparatus.

Assessment of the expression of LFA-3 was done using CD58-FITC (BD Bioscience, 555920), while the expression of Treg cells was based on number of helper cells CD4 with CD25 (FITC), HLA-DR (APC) (B.D Bioscience, San Jose, CA, USA) and HLA-DR (FITC) (35298).

To detect the expression of the required marker, 20 μl BD monoclonal antibodies was added to 100 μl whole blood in a tube, mixed thoroughly and incubated for 20 min in the dark room at 4–8 °C. Two millilitres FACS lysing solution (Becton Dickinson) was added and the tube was vortexed thoroughly. After a further 10 min of incubation in the dark room at 4–8 °C, the tubes were centrifuged at 3000 r.p.m. for 10 min, the supernatant discarded and the cells washed with phosphate-buffered saline (PBS) with 0.1% azide and centrifuged again at 400*g* for 10 min. A second wash was performed, and after the supernatant was discarded, 0.5 ml PBS was put in the tube and the specimen analysed. Using FACScan (Becton Dickinson) and the appropriate software (Cell Quest, Becton Dickinson), the population of lymphocytes was identified from forward and side scatter characteristics on dot plot profiles, with bright CD45 and analysed for fluorescence intensity using defined gates. Data collected were reported as either percentage of positive cells or mean fluorescence intensity (MFI) values.

## Statistics

Data was analysed using SPSS for Windows Release 15. A two-tailed *p* value of ≤ 0.05 was considered statistically significant. For descriptive statistics of qualitative variables, the frequency distribution procedure was run with calculation of the number of cases and percentages. For descriptive statistics of quantitative variables, the mean and standard deviation or the median and range were used to describe central tendency and dispersion as appropriate. Normality of the sample distribution of each continuous variable was tested with the Kolmogorov–Smirnov test. Association between categorical variables was tested by the chi-square test. Fisher’s exact test was used if the assumptions of the chi-square were violated. The independent sample *t* test was used to compare the means between two groups. One-way analysis of variance (ANOVA) was used to compare the means between more than two groups. For nonparametric analysis, the Mann–Whitney *U* test was used. ROC analysis was used to assess different markers; each of which to be a diagnostic tool.

## Results

In this study, thyroid disease was more common in females than males: 88% in HT, 53.3% in GD and 86.7% in CG. Patients in groups I, II and III presented with either nodular or diffuse thyroid swelling, and as a result, it was sometimes difficult to reach diagnosis until after full investigation of thyroid function, possibly even requiring the use of invasive techniques; diffuse thyroiditis was present in both HT and GD, while exophthalmos was a characteristic feature of GD only. Both HT and GD were in older when compared with the non-immune or control groups, as presented in Table 1.

**Table 1:** Demographic and clinical presentation of studied groups

		Hashimoto’s thyroiditis (group I) (*N* = 25)	Graves’ disease (group II) (*N* = 15)	Colloid goitre (group III) (*N* = 15)	Normal control (group IV) (*N* = 20)	*P*
Male		3 (12%)	7 (46.7%)	2 (13.3%)	9 (45%)	0.017**
Female		22 (88%)	8 (53.3%)	13 (86.7%)	11 (55%)	
Clinical	Normal	0 (0%)	0 (0%)	0 (0%)	20 (100%)	< 0.001***
	Subclinical	12 (48%)	2 (13.3%)	0 (0%)	0 (0%)	
	Nodular	4 (16%)	2 (13.3%)	12 (80%)	0 (0%)	
	Diffuse	9 (36%)	7 (46.7%)	3 (20%)	0 (0%)	
	Exophathalamous	0 (0%)	4 (26.7%)	0 (0%)	0 (0%)	
Age (mean ± SD)		37.04 ± 12.56	48.77 ± 14.4	36.26 ± 6.1	37.1 ± 9.1	0.005**

Data represented in number and percent. Quantitative data represented as mean. Test used is chi-square test

*SD* standard deviation

**Highly significant

***Extremely high significant

Thyroid markers were remarkably low in HT, while increases in FT3 and FT4 were seen in GD with marked suppression of TSH. CG showed nearly normal thyroid function. Lower expression of Treg cell harbouring CD4/CD25/HLA-DR was observed in both HT and GD, while higher expression was seen in the CG and control groups.

CD4/CD8 ratio was lower in AITD while normal in CG and control: HT (0.326 ± 0.92), GD (0.49 ± 0.19), CG (1.56 ± 0.26) and control (1.37 ± 0.45). CD4/CD8 ratio showed a highly significant difference between all groups; *p* = 0.002. This could imply its importance in the pathogenesis of thyroid disorders.

Higher expression of LFA-3 (CD58) was observed in AITD than in CG and control groups as illustrated in Table 2. Mean CD58 was 45.33 ± 12.79 in HT, 48.16 ± 11.27 in GD, 20.6 ± 4.93 in CG and 11.8 ± 1.82 in control with high significant differences between these groups (*p* < 0.001).

**Table 2:** Laboratory thyroid function testing and immunophenotyping in studied groups

	Hashimoto’s thyroiditis (group I) (*N* = 25)	Graves’ disease (group II) (*N* = 15)	Colloid goitre (group III) (*N* = 15)	Normal control (group IV) (*N* = 20)	*P*
Free T3	4.54 ± 2.52	14.33 ± 12.46	5.06 ± 0.82	5.16 ± 0.86	< 0.001***
Free T4	13.2 ± 4.85	45.17 ± 32.73	16.47 ± 3.56	17.72 ± 2.79	< 0.001***
TSH	11.64 ± 19.84	0.29 ± 0.71	2.1 ± 1.17	2.15 ± 1.04	0.008**
CD4	1.99 ± 0.51	1.56 ± 0.43	10.83 ± 2.82	10.8 ± 4.48	< 0.001***
CD8	6.3 ± 1.73	3.4 ± 1.06	7.21 ± 2.23	8.22 ± 1.54	< 0.001***
CD25	1.08 ± 0.53	1.08 ± 0.39	7.5 ± 1.31	6.6 ± 1.33	< 0.001***
HLA-DR	1.01 ± 0.43	1.3 ± 0.39	6.51 ± 1.57	5.79 ± 1.32	< 0.001***
CD58	45.33 ± 12.79	48.16 ± 11.27	20.6 ± 4.93	11.8 ± 1.82	< 0.001***

*CD* cluster differentiation, *HLA-DR* Human Leucocytic Antigen-Determing Region

Anti-thyroglobulin was significantly higher (*p* = 0.026) in HT than GD, CG and control groups, while anti-TPO was significantly (*p* < 0.001) higher in GD than in all other groups. Prolactin, white leucocyte count and absolute lymphocytosis showed no significant difference as presented in Table 3.

**Table 3:** Relation of thyroid autoantibody, prolactin hormone, and absolute lymphocytosis in different studied groups

	Hashimoto’s thyroiditis (group I) (*N* = 25)	Graves’ goitre (group II) (*N* = 15)	Colloid goitre (group III) (*N* = 15)	Normal control (group IV) (*N* = 20)	*P*
Anti-TG	191.83 ± 356.71	70.34 ± 50.7	18.39 ± 2.9	21.25 ± 7.03	0.026*
Anti-TPO	158 ± 147.03	763 ± 427.51	53 ± 31.66	48.15 ± 30.47	< 0.001***
Prolactin	253.79 ± 115.12	164.9 ± 109.59	257.24 ± 122.88	287.99 ± 162.83	0.05
WBC	7057.2 ± 2228.03	6932 ± 2057.8	8096.6 ± 2036.9	7159.5 ± 1712.2	0.363
ALC	4155.2 ± 2044.3	4596.6 ± 2081.7	3482.2 ± 2401.7	2911.5 ± 1000.2	0.05

*Anti-TG* anti-thyroglobulin, *Anti-TPO* anti-thyroperoxidase, *WBC* white leucocyte count, *ALC* absolute lymphocytes count

Mann–Whitney test for CD58 was used as differentiating marker between all studied groups and revealed a highly significant difference (*p* = 0.001) between HT and GD when compared with CG and with the control group, while no significant difference (*p* = 0.485) was detected between HT and GD (Table 4).

**Table 4:** Mann–Whitney Test for CD58, CD4/CD8 ratio, and NLR between different immunological thyroid disorders

Groups	CD58	CD4/CD8	N/L Ratio
Hashimoto’s versus Graves’	0.485	0.002	0.04
Hashimoto’s versus Colloid	0.001	0.001	0.665
Graves’ versus Colloid	0.001	0.001	0.071

Highly significant difference in CD4/CD8 ratio among all different thyroid disorder, while highly significant difference was observed for CD58 among Hashimoto’s versus Colloid, also Graves’ versus Colloid, whereas there was a significant difference in neutrophils/lymphocyte ratio among Hashimoto’s versus Graves’

Neutrophil/lymphocyte ratio revealed mild significant difference (*p* = 0.04) in HT compared with GD, while no significant difference was observed between HT or GD compared with CG or the control group.

**Fig. 2:**
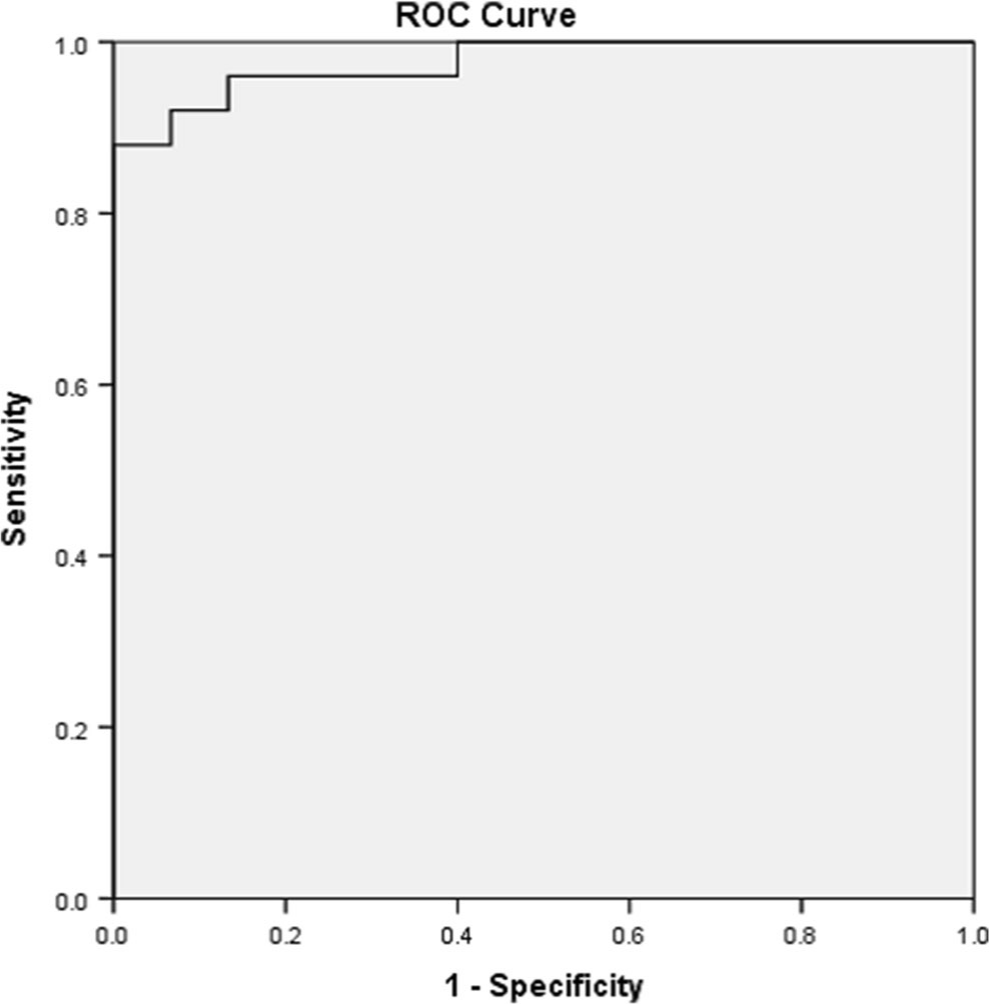
For CD58 with sensitivity 88% and specificity 100%, cut-off value more than or equal to 29.9 indicates Hashimoto’s disease, while lower value means Colloid disease

**Fig. 3:**
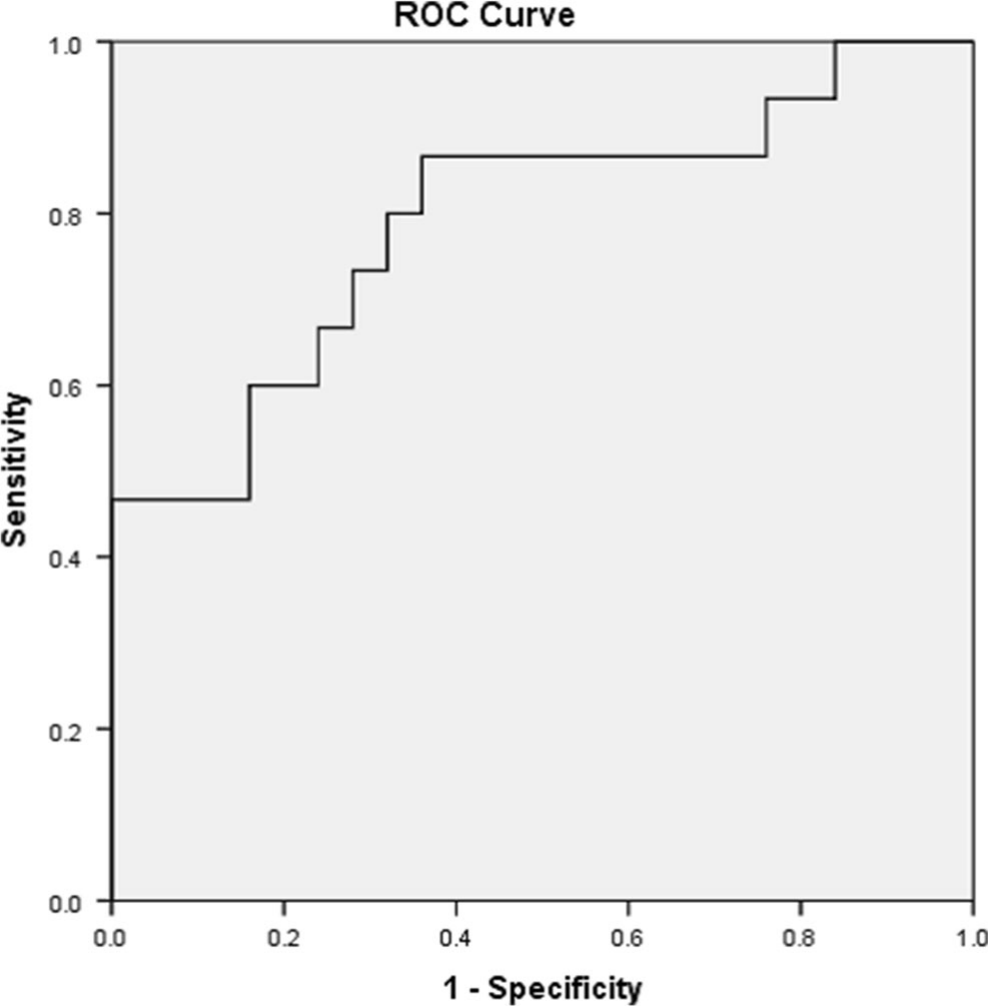
ROC curve for NLR (neutrophil lymphocyte ratio) with sensitivity 60% and specificity 87%. Cut-off value more than or equal to 0.5038 indicates Hashimoto’s disease, while lower value means Graves’ disease

ROC curve for CD58, with 88% sensitivity and 100% specificity, revealed that CD58 could be a differentiating marker between HT and CG with cut-off value 29.9 with higher values in HT and lower values in CG (Fig. 2). It could be also a differentiating marker between GD and CG, higher or equal to 29.84 in GD with lower values for CG with 100% sensitivity and specificity. However, ROC curve analysis of neutrophil/lymphocyte ratio with low sensitivity (60%) and specificity (87%) with cut-off 0.503 was recorded in HT and lower values in GD (Fig. 3).

## Discussion

Our study revealed lower expression of Treg cells in both AITD (HT and GD) when compared with the CG and control. This lower expression could be indicative of their role in the pathogenesis of autoimmune disorders and may explain the process by which autoantigens escape from self-tolerance as suggested (Nada and Hammouda 2014). However, (Fountoulakis et al. 2008) found increases in CD4+, CD25 and HLA-DR cells and concluded that compensatory expansion of these subpopulations of Treg occurred in an attempt to suppress the immune response. The number of Treg cells could be influenced by thymus output; however, their maintained state in the periphery is a dynamic process affected by genetic factors, proliferation rates of these cells and movement of these cells to inflammatory sites. Therefore, focusing research on Treg cells with regard to their value and importance in the pathogenesis of autoimmune diseases may help in the development of new therapeutic interventions (Teran et al. 2011).

The reduced expression of Treg cells found in our study could be explained as increased shift of Treg with expression of CD25+ into memory/effector T cell that enhance traffic towards inflamed tissue, where they exert their suppressive function in target organ as stated by (Dejaco et al. 2006)

Our findings were also in agreement with Mao et al. (2011) who explained that antigen presenting cells, mainly dendritic cells which present in peripheral and lymphoid organs, were polarised in untreated GD patients leading to a decrease in the number and regulatory capacity of Treg cells through induction of apoptosis. (Mao et al. 2015)

Furthermore, patients with severe inflammation and immune dysregulation or polyendocrinopathy have depletion of CD25+CD4+ T cell developing a variety of autoantibodies (Wing and Sakaguchi 2010).

Additionally, the absence of CD4+CD25+ Treg cells can lead to organ-specific and non-specific autoimmune disease such as gastritis, thyroiditis and systemic lupus erythromatosis, while application of these cells can prevent or delay these diseases (Toubi 2008).

Several researchers revealed that inadequate Treg cell expression may be involved in contact-dependent suppression or failure to produce soluble factors needed in its mechanism of suppression (Buckner 2010).

In light of our study and other studies, targeting Treg cells will change the future of treatment of immune thyroid disorders by improving suppressive ability of these cells that are implicated in many autoimmune disorders.

Another T-lymphocyte subset, CD8+ T cells, was decreased in our study in both HT and GD groups when compared to controls. This finding matched with Xia et al. (2006) who found imbalance in Th1/Th2 in Graves’ ophthalmopathy. These suppressive cells play an important role in pathogenesis of autoimmune disorders as they can downregulate autoimmune response (Xia et al. 2006). However, our results disagree with Bonnyns et al.’s (Bonnyns et al. 1983) findings. The difference in expression of CD8 T cells may be due to different analysis techniques: advanced FACScan versus flow cytometry in our study.

In our study, suppression of both T-helper and T-suppressor cells was greater in AITD when compared with non-immune thyroid diseases or healthy controls, with downregulation of the CD4/CD8 ratio. This characterisation of CD4/CD8 T cells ratio was used to provide a more comprehensive picture. Our finding of significantly decreased CD4/CD8 ratio is mismatched with (Gessl et al. 1995) in the HT group, and they also reported a significantly increased ratio in Graves’ disease, which is also contrary to our finding. This disparity could be explained by difference in the population studies as genetic factors may play a role in determination of CD4/CD8 T cell ratio in humans with at least some of the responsible genes that are located in the HLA complex as explained (Ferreira et al. 2010).

Our study revealed enhanced highly significant increase in CD58 (leucocyte function adhesion-3) in autoimmune thyroid groups compared the control group. Interestingly, it was also increased in the CG group compared with the control.

T-lymphocytic adherence to thyroid cells by LFA-3 and other adhesion molecules results in stimulation of thyroid proliferation and goitre formation (Arao et al. 2000). Higher expression of LFA-3 in our study could be explained by its role in active presentation of autoantigen to APC with co-stimulatory signals which could play a part in the initial step of the autoimmune process. This finding may reflect important value of this marker as a therapeutic target to stop the immune process. In addition, its higher value in colloid goitres points to the fact that development of an autoimmune thyroid disease still requires not only LFA-3 to be presented on APC but also co-stimulatory signals, which are not present in colloid goitres.

ROC curve for CD58 with 88% sensitivity and 100% specificity revealed that CD58 could be a differentiating marker between AITD and CG with cut-off value 29.9 with higher values in HT, and equal to, or higher than, 29.84 in GD relative to CG with 100% sensitivity and specificity. This raises important value of this marker as a differentiating test between immune thyroid disorders and colloid disease.

Recently, several articles have focussed on the importance of neutrophil/lymphocyte ratio as a simple and applicable finding from routine cell counting as predictor for differentiating benign from malignant thyroid diseases. Few studies about this predictor have been documented in immunological disorders, so we tried to assess the value of this marker in immunological and non-immunological thyroid disorders. There are many cut-offs in different studies done on malignant thyroid disorders but not in immunological diseases (Kocer et al. 2015). ROC analysis of our data suggested that values greater or equal to the cut-off values, 0.5038, indicate HT with sensitivity 60% and specificity 87%, whereas lower values indicate GD.

In summary, assessment of different markers was done in this study to help in the screening of different thyroid immunological diseases which need invasive procedures, such as needle aspiration and biopsy to be fully diagnosed. CD58, CD4/CD8 ratio could help in differentiation, while NLR may have little diagnostic value but may be important in malignancy disorders, so further studies are required to validate this marker as simple routine investigation to complete final diagnosis.

## Conclusion

Downregulation of Treg cells with higher expression of LFA-3 in both Hashimoto’s and Graves’ disease groups could suggest their role in pathogenesis of these autoimmune diseases and may open therapeutic hope for those patients. CD58 could also be used as differentiating marker between autoimmune thyroid diseases and non-immune thyroid diseases. We need extended study in large cohort group to validate this finding.

## References

[CR1] Weetman AP (2003). Autoimmune thyroid disease: propagation and progression. Eur J Endocrinol.

[CR2] Fountoulakis S, Tsatsoulis A (2004). On the pathogenesis of autoimmune thyroid disease: a unifying hypothesis. Clin Endocrinol.

[CR3] Ehlers M, Thiel A, Bernecker C, Porwol D, Papewalis C, Willenberg HS, Schinner S, Hautzel H, Scherbaum WA, Schott M (2012). Evidence of a combined cytotoxic thyroglobulin and thyroperoxidase epitope-specific cellular immunity in Hashimoto` thyroiditis. J Clin Endocrinol Metab.

[CR4] McLachlan SM, Nagayama Y, Pichurin PN, Mizutori Y, Chen CR, Misharin A (2007). The link between Graves’ disease and Hashimoto’s thyroiditis: a role for regulatory T cells. Endocrinology.

[CR5] Dantus LH (2008). Environmental factors and autoimmune thyroditis. Nat Clin Pract Endocrinol Metab.

[CR6] Tomer Y (2010). Genetic susceptibility to autoimmune thyroid disease: past, present, future. Thyroid.

[CR7] Armengol MP, Sabater L, Fernadez M, Ruiz M, Alonso N, Otero MJ (2008). Influx of recent thymic emigrants into autoimmune thyroid disease glands in humans. Clin Exp Immunol.

[CR8] Miyara M, Sakaguchi S (2007). Natural regulatory T cells: mechanisms of suppression. Trends Mol Med.

[CR9] Sakaguchi S (2005). Naturally arising Foxp3-expressing CD25+CD4+ regulatory T cells in immunological tolerance to self and non-self. Nat Immunol.

[CR10] Endharti AT, Rifa’i M, Shi Z, Fukuoka Y, Nakahara Y, Kawamoto Y (2005). Cutting edge: CD8+CD122+ regulatory T cells produce IL-10 to suppress IFN-gamma production and proliferation of CD8+ T cells. J Immunol.

[CR11] Curotto de Lafaille MA, Lafaille JJ (2009). Natural and adaptive foxp3+ regulatory T cells: more of the same or a division of labor?. Immunity.

[CR12] Thornton AM, Korty PE, Tran DQ, Wohlfert EA, Murray PE, Belkaid Y, Shevach EM (2010). Expression of Helios, an Ikaros transcription factor family member, differentiates thymic-derived from peripherally induced Foxp3+ T regulatory cells. J Immunol.

[CR13] Wingren AG, Parra E, Varga M, Kalland T, Sjogren HO, Hdlund G (1995). T cell activation pathways: B7, LFA-3, and ICAM-1 shape unique T cell profiles. Crit Rev Immunol.

[CR14] Wang JH, Smolyar A, Tan K, Liu JH, Kim M, Sun ZY (1999). Structure of a heterophilic adhesion complex between CD2 and CD58 (LFA-3) counter receptors. Cell.

[CR15] Baykan H, Cihan YB, Ozyurt K (2015). Roles of white blood cells and subtypes as inflammatory markers in skin cancers. Asian Pac J Cancer Prev.

[CR16] Ozyalvcli G, Yesil C, Kargi E, Kizildage B, Kilitci A, Yilmaz F (2014). Diagnostic and prognostic importance of the neutrophils lymphocyte ratio in breast cancer. Asian Pac J Cancer Prev.

[CR17] Nada AM, Hammouda M (2014). Immunoregulatory T cells, LFA-3 and HLA-DR in autoimmune thyroid diseases. Indian J Endocrinol Metab.

[CR18] Fountoulakis S, Vartholomatos G, Kolaitis N, Frillingos S, Philippou G, Tsatsoulis A (2008). HLA-DR expressing peripheral T regulatory cells in newly diagnosed patients with different forms of autoimmune thyroid disease. Thyroid.

[CR19] Teran R, Mitre E, Vaca M, Erazo S, Oviedo G, Hübner MP, Chico ME, Mattapallil JJ, Bickle Q, Rodrigues LC, Cooper PJ (2011). Immune system development during early childhood in tropical Latin America: evidence for the age-dependent down regulation of the innate immune response. Clin Immunol.

[CR20] Dejaco C, Duftner C, Grubeck –LB, Schirmer M (2006). Imbalance of regulatory T cells in human autoimmune diseases. Immunology.

[CR21] Mao C, Wang S, Xiao Y, Xu J, Jiang Q, Jin M (2015). Impairment of regulatory capacity of CD4+CD25+ regulatory T cells mediated by dendritic polarization and hyperthyroidism in Grave’s disease. J Immunol.

[CR22] Wing K, Sakaguchi S (2010). Regulatory T cells exert checks and balances on self-tolerance and autoimmunity. Nat Immunol.

[CR23] Toubi E (2008). Targeting T regulatory cells in autoimmune diseases. Isr Med Assoc J.

[CR24] Buckner JH (2010). Mechanisms of impaired regulation by CD4+CD25+FOXP3+ regulatory T cells in human autoimmune diseases. Nat Rev Immunol.

[CR25] Xia N, Zhou S, Liang Y, Xiao C, Shen H, Pan H, Deng H, Wang N, Li QQ (2006). CD4+T cells and the Th1/Th2 imbalance are implicated in the pathogenesis of Grave’s ophthalmopathy. Int J Mol Med.

[CR26] Bonnyns M, Bentin J, Devetter G, Duchateau J (1983). Heterogeneity of immunoregulatory T cells in human thyroid autoimmunity: influence of thyroid status. Clin Exp Immunol.

[CR27] Gessl A, Wilfing A, Agis H, Steiner G, Czernin S, Boltz-Nitulescu G (1995). Activated naive CD4+ peripheral blood T cells in autoimmune thyroid disease. Thyroid.

[CR28] Ferreira MA, Mangino M, Brumme CJ, Zhao ZZ, Medland SE, Wright MJ (2010). Quantitative trait loci for CD4:CD8 lymphocyte ratio are associated with risk of type 1 diabetes and HIV-1 immune control. Am J Hum Genet.

[CR29] Arao T, Morimoto I, Kakinuma A, Ishida O, Zeki K, Tanaka Y, Ishikawa N, Ito K, Eto S (2000). Thyrocyte proliferation by cellular adhesion to infiltrating lymphocytes through the intercellular adhesion molecule-1/lymphocyte function –associated antigen-1 pathway in Grave’s disease. J Clin Endocrinol Metab.

[CR30] Kocer D, Karakukcu C, Karaman H, Gokay F, Bayram F (2015). May the neutrophils /lymphocyte ratio be a predictor in the differentiation of different thyroid disorders?. Asian Pac J Cancer Prev.

